# Canine Cognitive Dysfunction and Alzheimer’s Disease – Two Facets of the Same Disease?

**DOI:** 10.3389/fnins.2019.00604

**Published:** 2019-06-12

**Authors:** Sonja Prpar Mihevc, Gregor Majdič

**Affiliations:** ^1^Veterinary Faculty, Institute for Preclinical Sciences, University of Ljubljana, Ljubljana, Slovenia; ^2^Medical Faculty, Institute for Physiology, University of Maribor, Maribor, Slovenia

**Keywords:** brain, neurodegeneration, canine cognitive dysfunction, Alzheimer’s disease, amyloid beta, TAU, animal model, treatment

## Abstract

Neurodegenerative diseases present a major and increasing burden in the societies worldwide. With aging populations, the prevalence of neurodegenerative diseases is increasing, yet there are no effective cures and very few treatment options are available. Alzheimer’s disease is one of the most prevalent neurodegenerative conditions and although the pathology is well studied, the pathogenesis of this debilitating illness is still poorly understood. This is, among other reasons, also due to the lack of good animal models as laboratory rodents do not develop spontaneous neurodegenerative diseases and human Alzheimer’s disease is only partially mimicked by transgenic rodent models. On the other hand, older dogs commonly develop canine cognitive dysfunction, a disease that is similar to Alzheimer’s disease in many aspects. Dogs show cognitive deficits that could be paralleled to human symptoms such as disorientation, memory loss, changes in behavior, and in their brains, beta amyloid plaques are commonly detected both in extracellular space as senile plaques and around the blood vessels. Dogs could be therefore potentially a very good model for studying pathological process and novel treatment options for Alzheimer’s disease. In the present article, we will review the current knowledge about the pathogenesis of canine cognitive dysfunction, its similarities and dissimilarities with Alzheimer’s disease, and developments of novel treatments for these two diseases with a focus on canine cognitive dysfunction.

## Introduction

Neurodegenerative diseases such as Alzheimer’s disease (AD), Parkinson’s disease, Huntington’s disease, frontotemporal dementia, amyotrophic lateral sclerosis, and others are a major growing public health problem associated with aging, as aging is the greatest risk factor for neurodegeneration. The global number of people living with dementia more than doubled from 1990 to 2016 (GBD 2016 Dementia Collaborators, 2019). These diseases now affect nearly 50 million individuals, and the incidence is projected to triple by 2050. AD and other dementias are associated with alterations in cell type-specific function, including gliosis, neuronal dysfunction and cell death. The pathognomic cause is the condensation of certain proteins into insoluble aggregates and this aggregates damage vulnerable neurons and glial cells. These inclusions of misfolded proteins build up in the brain during normal aging and during the progression of adult-onset dementias.

Neurodegenerative diseases do not occur spontaneously in laboratory mice and rats, but do occur in several other mammalian species. Studies of neurodegenerative diseases in animals have shown strong similarities between cognitive dysfunction in dogs and human AD and between AD in humans and most other primates (Cummings et al., 1993; Cummings and Cotman, 1995; Dodart et al., 2002; Chambers et al., 2011; Braidy et al., 2015; Schütt et al., 2016). Canine cognitive dysfunction (CCD), also known as canine cognitive dysfunction syndrome (CDS) or canine dementia affects up to 60% of older dogs, mostly dogs older than 11 years (Fast et al., 2013b). The prevalence of CCD does not differ between breeds (Salvin et al., 2010) and there are no breed specific differences in clinical signs or pathology of the disease. However, as larger dog breeds have shorter life span than the smaller ones (Greer et al., 2007), clinical signs of CCD are more often observed and reported in smaller dogs (Vite and Head, 2014; Schmidt et al., 2015). Due to its similarity with AD, CCD is extremely interesting as a model for human disease. CCD is also a major problem for health of older dogs, and is thus also interesting from the point of view of the development of new veterinary diagnostic procedures/markers and medicines for treating this disease in dogs.

In dogs and humans, dementia often affects cerebral gyri (cerebral atrophy) and shows as widening of sulci together with ventricular enlargement (Borràs et al., 1999; Toepper, 2017). Neuronal loss and cortical atrophy has been described in several brain regions including cortex, hippocampus and parts of the limbic system in cognitively impaired dogs (Tapp et al., 2004; Siwak-Tapp et al., 2008), similarly to human brain affected by AD (West et al., 2000). In human patients, AD has a preclinical stage with the clinical symptoms not yet evident but with pathological changes in the brain already present, which is followed by mild cognitive impairment stage which gradually culminates in dementia due to AD, with severe cognitive and functional decline (Bature et al., 2017). The onset of AD, detected clinically at the mild cognitive impairment stage, is characterized by a subtle decline in memory functions, followed by changes in personality, deterioration of language functions and eventually motor dysfunction (Braak and Braak, 1997). These deficits in cognition and behavior are mirrored in the AD-related regional brain destruction. Similarly to these signs in humans, the most obvious signs of CCD, deduced by standard cognition tests, are loss of memory, poor or completely lost sense of orientation, changes in behavior and confusion (Bain et al., 2001). Most common symptoms include disorientation, anxiousness, dogs get easily scared, do not recognize their owner, become aggressive or apathetic, have difficulty controlling the secretion of body fluids, change circadian rhythms and others.

## Neuropathological Features of Neurodegenerative Diseases

### Role of Protein Aggregates in Neurodegenerative Diseases

Protein aggregation is an established pathogenic mechanism in AD, although little is known about the initiation of this process *in vivo*. As human brain research largely depends on the results of postmortem studies, an insight into the early stages of the disease, when protein aggregates are most likely to occur, is difficult. Novel and better animal models would therefore be very helpful to study the progress of AD in humans. The clinical course of the disease in humans could be monitored by brain imaging (CT, MRI). The exact connection between protein aggregates, such as extracellular amyloid-β (Aβ) plaques and intracellular neurofibrillary tangles (NFTs), the latter composed of hyperphosphorylated microtubule associated protein (TAU), in the brains of patients with AD, and neurodegeneration is therefore still unknown. It does seem that the AD is caused by the accumulation of amyloid plaques and neurofibrillary fibers, although this is not absolutely confirmed and generally accepted (Medeiros et al., 2011). Stronger evidence that aggregates do trigger neurodegeneration (the so-called amyloid hypothesis) and are not just a consequence of the neurodegeneration, is provided by mutations in the gene for the amyloid beta precursor protein (APP) present in some human patients with AD (Selkoe, 1991; Hardy and Higgins, 1992; Hardy and Selkoe, 2002; Lansbury and Lashuel, 2006). Similarly, amyloid plaques are present in different brain regions in dogs with CCD clinical symptoms, and they can be present even before clinical signs of this disease become obvious.

In general, age-related neurodegenerative disorders are complex and multifaceted pathologies, wherein the formation of large aggregates and/or high concentrations of toxic proteins prevent the proper function of neuronal cells, leading to ischemia and eventually tissue removal. The spread of AD pathology follows a characteristic topographic pattern, different for the two proteins involved in the pathology, Aβ and TAU (Brettschneider et al., 2015). TAU aggregates first develop in the locus coeruleus, then in entorhinal cortex followed by hippocampus and neocortex. Aβ plaques firstly appear in the neocortex, and later in allocortical, diencephalic and basal ganglia structures and in the brainstem (Brettschneider et al., 2015; Jucker and Walker, 2018). These aggregates spread in a prion-like manner, forming intracellular and extracellular deposits. TAU, Aβ, and also Parkinson disease associated α-synuclein, exist in different conformational variants (‘strains’) that show seeding properties and exert different levels of neurotoxicity, which could be the source of heterogeneity of neurodegenerative diseases (Aguzzi, 2014). The seeding potential of these aggregates was recently substantiated by demonstrating propagation of structural variants of Aβ in their distinct conformations through template-directed folding of naïve Aβ peptides (Condello and Stöehr, 2018). Prion-like transmission and seeding has been also observed for TAU (DeVos et al., 2018). Similar mechanism may be involved in young, pre-depositing APP transgenic mice which developed cerebral β-amyloidosis and associated pathology after being intracerebrally injected with Aβ-containing brain extracts from human patients with AD (Meyer-Luehmann et al., 2006; Langer et al., 2011). Interestingly, even intraperitoneal inoculation with Aβ extracts induced β-amyloidosis in the brains of APP transgenic mice after prolonged incubation times (Eisele et al., 2010). Likewise, soluble oligomers from blood and cerebrospinal fluid (CSF) of an aged dog with CCD were neurotoxic to human neuroblastoma cell line, and canine CSF derived Aβ induced the *in vitro* aggregation of synthetic human Aβ peptides (Rusbridge et al., 2018). Spreading of misfolded Aβ oligomers in a prion-like mechanism might also exploit exosomes, which can seed Aβ not only as vehicles for the neuron-to-neuron transfer (Zheng et al., 2017; Sardar Sinha et al., 2018) but also between distant brain regions (i.e., from the dentate gyrus to other regions of hippocampus and to the cortex) (Zheng et al., 2017).

### The Neuropathology in the Brain Parenchyma

**FIGURE 1:**
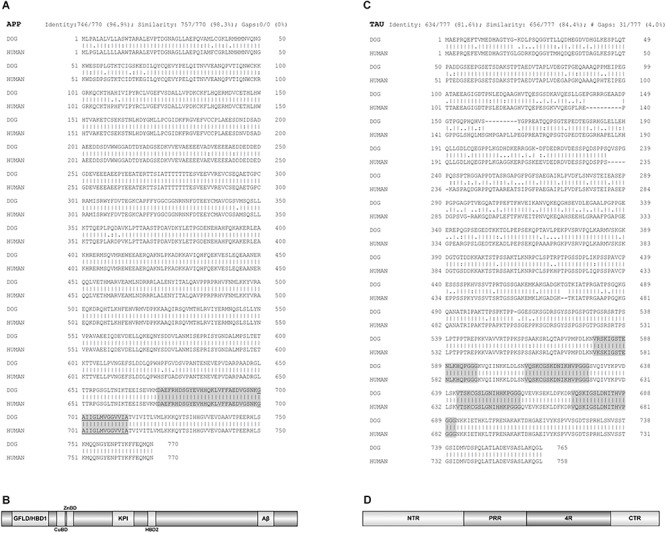
Protein sequences alignments between dog and human amyloid-beta precursor protein (APP) and TAU. **(A)** The alignment of the longest canine APP isoform (APP-770) sequence with protein sequence of human APP. Aligned sequences share 96.9% amino acid identity and 98.3% similarity. Of note, the sequence identity of canine APP-695 isoform, the predominant APP isoform in the canine brain, and human APP is 87.5%. Highlighted is Aβ domain and underlined the membrane bound part of this domain. **(B)** Domain structure of APP. Isoform APP-770 is depicted. HBD1/GFLD, heparin binding domain 1/growth factor like domain; CuBD, copper binding domain; ZnBD, zinc binding domain; KPI, Kunitz-type protease inhibitor domain (not present in APP-695); Aβ, amyloid beta domain, the latter anchors APP in the cell membrane. The α-, β-, and γ-secretase cleavage sites are directly adjacent or inside the Aβ domain region, which is also most mutation prone, thus enabling alternative cleavage of APP and its divergent aggregation propensities. **(C)** Alignment of the longest TAU protein isoform, out of six existing, of dog and human TAU. Aligned TAU sequences share 81.6% amino acid identity and 84.4% similarity. Highlighted are four microtubule-binding regions (4R). **(D)** Domain structure of TAU. The basic organization is shown. NTR, N-terminal region; PRR, proline-rich region; 4R, four microtubule-binding regions (some isoforms have three); CTR, C-terminal region. The PRR and 4R domains are subject of most posttranslational modifications, while the CTR enables interactions with microtubules and plasma membrane. EMBOSS Needle pairwise sequence alignment (Madeira et al., 2019) was performed. A line (|) indicates positions which have a single, fully conserved residue. A colon (:) indicates conservation between groups of strongly similar properties and a dot (.) indicates conservation between groups of weakly similar properties. The domain organization was depicted by software illustrator of biological sequences (IBS) (Liu et al., 2015). Figure made by the authors.

The most prominent neuropathological signs of AD are accumulation of Aβ in a form of extracellular plaques in the brain parenchyma and also in the walls of blood vessels (cerebral amyloid angiopathy, CAA), and abnormally phosphorylated protein TAU that accumulates in NFTs (Braak and Braak, 1991; Nelson et al., 2012). These pathological features are believed to cause cognitive and behavioral changes. The amino acid sequence of APP and of the enzymes involved in the processing of Aβ peptides from APP, are highly homologous between humans and dogs (Johnstone et al., 1991; Sarasa and Pesini, 2009; Sarasa et al., 2010). Major canine APP isoforms are APP-770, APP-751, APP-714, and APP-695 (Sarasa et al., 2010). The alignment of the longest canine amyloid-beta precursor protein isoform (APP-770) sequence with protein sequence of human amyloid-beta precursor protein show 96.9% amino acid identity and 98.3% similarity (Figure 1A), making them almost identical. The expression patterns of canine APP isoforms are almost similar to the patterns previously detected for human APP isoforms (Yasojima et al., 2001; Sarasa et al., 2010). APP is a single-pass transmembrane protein with a large extracellular domain and a short cytoplasmic tail. The domain structure of APP-770 is shown in Figure 1B, the neuronal isoform APP-695 lacks the KPI domain (Müller et al., 2017). In contrary to APP, there is a difference in the TAU protein sequence between dogs and humans. Figure 1C shows the longest TAU isoform alignment between dog and human protein sequences with 84% similarity. Interestingly, the four microtubule-binding regions (4R) and the C-terminal regions (Figures 1C,D) are identical.

Amyloid-β was found to be present in the form of insoluble plaques in the area of the cerebral cortex in humans and dogs, and cognitive impairment in elderly dogs was in some studies strongly associated with the accumulation of Aβ in the brain (Cummings et al., 1993; Cummings and Cotman, 1995; Pugliese et al., 2006; Rofina et al., 2006; Barnes et al., 2016). However, in two other studies there was no significant correlation between the amount of Aβ brain load and CCD symptoms (Chambers et al., 2011; Ozawa et al., 2016) so the clear connection between Aβ load in the brain and clinical signs of CCD has not been firmly established yet. Although Aβ plaques are generally detected as extracellular deposits of diffuse and neuritic plaques in humans and dogs, some studies also reported the presence of Aβ deposits inside individual neurons in the canine brain (Cummings et al., 1993; Yu et al., 2011). Similarly, intraneuronal Aβ accumulation in human AD brains has been reported by many groups (reviewed in detail by Gouras et al., 2010).

**FIGURE 2:**
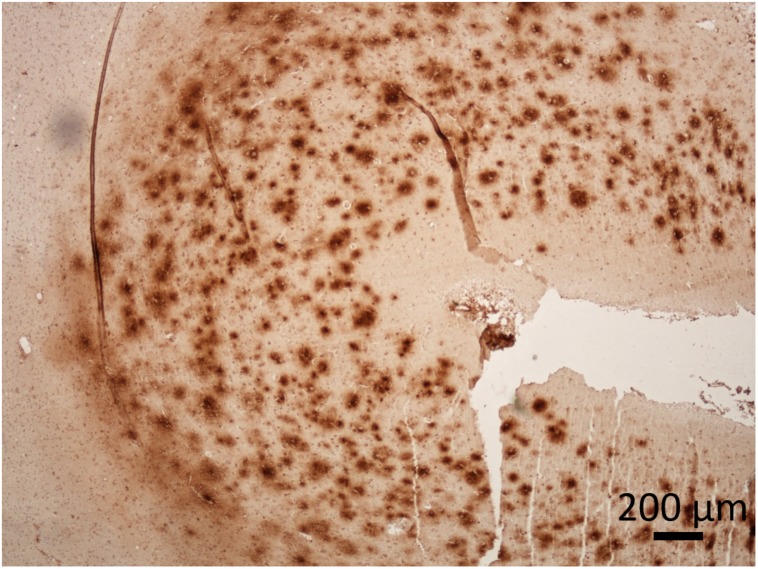
Presence of Aβ in the cerebral cortex of a dog with CCD. The dense plaques detected in superficial cortical layers and diffuse plaques in deeper layers of prefrontal cortex. The dog was 17-years-old of a mixed breed. Immunoperoxidase staining with antibodies against Aβ (purified anti-β-Amyloid, 17-24 Antibody, BioLegend, #800701) with diaminobenzidine (DAB) as chromogen (brown), counter stained with hematoxylin. Original microphotograph made by the authors.

In dogs, formation and maturation of Aβ deposits was observed by immunostaining throughout the canine cortical gray matter layers in a four-stage distribution, which is also characteristic for human AD, and this, according to some studies, correlates with the severity of cognitive deficit in the dog (Bosch et al., 2012) and varies as a function of age and size (weight) in companion dogs (Rofina et al., 2006; Schmidt et al., 2015). The Aβ load was higher in small and medium size dogs, which can be explained by the longer life span of smaller dogs and thus longer time necessary to accumulate these deposits (Schmidt et al., 2015). Most of the studies, on CCD brains, were made on geriatric dogs of different breeds and sizes, hence in this review the pathology is mainly described without further specifications of individual dogs characteristics. The canine prefrontal cortex is normally the site of disease onset (Figure 2), which spreads progressively to the parietal, entorhinal and occipital cortices, and lesions in certain brain regions correlate with certain behavioral deficits, as shown in Table 1. In separate studies amyloid plaques of mainly diffuse type were detected in several regions of canine brain, in the frontal and temporal cortex (Ishihara et al., 1991; Colle et al., 2000; Pugliese et al., 2006; Schmidt et al., 2015; Ozawa et al., 2016; Schütt et al., 2016; Smolek et al., 2016), in entorhinal cortex and hippocampus (Cummings et al., 1993; Colle et al., 2000; Yu et al., 2011; Schmidt et al., 2015; Borghys et al., 2017) and parietal cortex (Yu et al., 2011). Hippocampal deposits of a subspecies of pyroglutamyl Aβ, the highly neurotoxic pE3AA, were also detected in demented dogs (Schmidt et al., 2015). These plaques were more abundant in small and medium dogs (Schmidt et al., 2015). Studies further suggest that earlier assembly states of Aβ, such as oligomers and protofibrils, may be neurotoxic in the dog’s brain (Head et al., 2010). The levels of Aβ soluble oligomers in the CSF correlated inversely with Aβ load in the CCD affected beagle brains (Head et al., 2010). Precipitates from the CSF of demented Samoyed dog with CCD acted highly neurotoxic in *in vitro* tests and were more neurotoxic than the synthetic oligomeric and fibrillary forms of Aβ (Rusbridge et al., 2018).

**TABLE 1:** Affected brain regions and underlying cognitive deficits in dogs.

**Brain region**	**Pathology**	**Cognitive decline**	**References**
Prefrontal cortex	Aβ	Executive function, behavioral changes [cognitive performance, loss of previously learned behaviors (e.g., house soiling)], motor skills, attention, emotions and impulse control (fearfulness, aggression, stereotypic pacing or circling)	Bosch et al.,2012
Frontal cortex	Aβ	Changes in executive functions (inhibitory control and complex working memory)	Ishihara et al., 1991; Schmidt et al., 2015; Smolek et al.,2016
Parietal cortex	Aβ	Sensory association, learning and memory	Yu et al., 2011; Nešić et al.,2017
Entorhinal cortex	Aβ, NFTs^*^	Visual learning, memory	Schmidt et al., 2015; Smolek et al.,2016
Occipital cortex	Aβ	Learning and memory (visual association area, visual cortex)	Martin et al.,2011
Temporal cortex	Aβ, NFTs^*^	Visual memory (facial recognition), emotions	Smolek et al.,2016
Hippocampus	Aβ, NFTs^*^	Changes in sleep–wake cycles, appetite control, complex working memory	Colle et al., 2000; Pugliese et al., 2006; Yu et al., 2011; Schmidt et al., 2015; Smolek et al.,2016
Cerebral cortex (not further specified)	Aβ	Disorientation, decrease in social interactions, changes in sleep–wake cycles, loss of prior housetraining, increased anxiety, changes in level of activity	Colle et al., 2000; Pugliese et al., 2006; Yu et al.,2011
Cerebral capillaries and arteries	Aβ - CAA	Lower perceptual speed and episodic memory	Ishihara et al., 1991; Colle et al., 2000; Yu et al.,2011
Meningeal blood vessels	Aβ - CAA		Ishihara et al., 1991; Borràs et al., 1999; Colle et al., 2000; Yu et al., 2011; Nešić et al.,2017

^*^NTFs are rarely detected in CCD.

Besides Aβ plaques, recruitment and activation of astrocytes and microglial cells has been noticed in dogs with CCD (astrocytosis, microgliosis) (Smolek et al., 2016; Rusbridge et al., 2018) along with astrocyte hypertrophy (Borràs et al., 1999). Furthermore, the levels of pathological Aβ in the canine prefrontal cortex were positively correlated with age but neither with the severity of cognitive deficits (Gómez-Pinedo et al., 2016) nor the neuronal cell loss (Cummings et al., 1993). Neprilysin (NEP) mRNA, coding for a pivotal Aβ-degrading protein, was poorly expressed in the prefrontal cortex of aged dogs with CCD (Canudas et al., 2014), similar to human AD brain, where areas with higher Aβ aggregation express lower levels of NEP (Reilly, 2001).

Aged dogs with cognitive impairment exhibit degeneration of noradrenergic neurons, which correlates with higher levels of Aβ deposits in the prefrontal cortex (Insua et al., 2010). Likewise, in human AD, locus coeruleus degeneration and loss of cortical noradrenergic neurons occurs already at early stage (Kelly et al., 2017). The activity of the canine cholinergic system also declines with age (Araujo et al., 2005b). Number of basal forebrain cholinergic neurons was significantly reduced in aged cognitively impaired dogs in comparison to aged cognitive unimpaired and young dogs, but unlike for noradrenergic neurons, this reduction of cholinergic neurons did not correlate with the extent of Aβ cortical load (Insua et al., 2012). As in CCD, cholinergic neurons located in the basal forebrain, including the neurons that form the nucleus basalis of Meynert, are usually lost in AD (Whitehouse et al., 1981). In human patients, the cholinergic neurons appear most vulnerable to Aβ pathology, followed by glutamatergic and GABAergic neurons (Bell et al., 2006). The synaptic loss correlates with the disease progression and destruction of cholinergic neurons contributes to memory and attention deficits in AD (Terry et al., 1991). These alterations in neurotransmitter systems, with reduced neuronal and synaptic function have also been observed in dogs (Landsberg et al., 2012) and result in clinical symptoms of CCD. The employment of the canine model to examine the effect of the cholinesterase inhibitors in treatment of CCD is described in details under “Treatment and drug development”.

TAU protein, another important factor in neurodegenerative diseases, is encoded by *MAPT* (microtubule associated protein tau) gene. Mutations in this gene have not been linked to AD, but cause a familial form of frontotemporal dementia (Olszewska et al., 2016). However, in human AD, intraneuronal NFTs composed of hyperphosphorylated TAU and misfolded insoluble TAU protein aggregates and extracellular Aβ inclusions are both present and necessary for the diagnosis of the disease. In AD NFTs form initially in the locus coeruleus, then entorhinal cortex and further progress to the hippocampus, anterior cingulate cortex, visual association area and finally to the primary visual cortex in the occipital lobe (Ossenkoppele et al., 2016; Hoenig et al., 2018). Interestingly, TAU neurofibrillary inclusions were only rarely identified in canine brain, for instance only in one dog in one study (Smolek et al., 2016) and in three dogs in another study (Schmidt et al., 2015). Increased phosphorylation of TAU was observed at some amino acid sites in canine brain, although no study so far confirmed the presence of vast mature NFTs deposits as are typically observed in human AD. In one study, cytoplasmic deposits of phosphorylated TAU (pTAUThr181) were detected in the prefrontal cortex, but no NFTs were observed (Pugliese et al., 2006). Smolek et al. (2016) detected increased presence of phosphorylated TAU protein (pTAU) in synaptosomes of demented dogs. This could suggest that dementia in dogs might be partially caused by the weakening of the synaptic function, caused by pTAU, and not by the toxic effects of NFTs. This is further supported by the increase in TAU hyperphosphorylation in individual cortical neurons and by pTAU subcellular distribution shift from perinuclear to granular cytoplasmic and nuclear, which correlates with dog’s age (Pugliese et al., 2006). Expression of pTAUSer396 and accumulation of ubiquitin were also significantly increased in the parietal cortex and dorsal part of the hippocampus in old dogs when compared to expression in humans (Pugliese et al., 2006; Yu et al., 2011). Specifically, pTAUSer396 expressing astrocytes and neurons with co-localization of pTAUSer396 and ubiquitin were also observed in the parietal cortices and hippocampi of dogs with CCD (Yu et al., 2011). However, cytoplasmic aggregates of normally predominately nuclear proteins, TAR DNA-binding protein (TDP-43) and fused in sarcoma (FUS), which are strongly associated with frontotemporal dementia and amyotrophic lateral sclerosis in human patients, were not detected in the canine brain with CCD (Smolek et al., 2016).

Neurodegenerative diseases occur spontaneously in other domestic animals, especially in cats (Chambers et al., 2015), although brain pathology in these other species is much less studied. In the hippocampi of naturally aged domestic cats, Aβ accumulation, NFTs formation and neuronal loss were observed (Chambers et al., 2015) and this might contribute to cognitive decline in this species, although this has not yet been firmly established. Cognitive deficit may occur in the horse, as clinical signs similar to cognitive impairment have been observed, but no detailed studies focusing on age-related neurologic aberrances have been conducted so far. In one study, TAU protein was shown to be present in equine hippocampal neurons, but no NFTs were detected (Capucchio et al., 2010). Some horses develop equine motor neuron disease, but none have been associated with pathological accumulation of dementia related proteins (El-Assaad et al., 2012; Youssef et al., 2016). Interestingly, a recent study negated the belief that abnormally behaving stereotypic horses (*Equus caballus*) are cognitively impaired (Briefer Freymond et al., 2018).

Neurodegenerative diseases most likely occur in many other mammalian species, but there are very limited reports about these. The reports are focusing on the presence of Aβ deposits and phosphorylated TAU and/or NFTs, which are detected post mortem, not describing the cognitive deficit, this is of course more difficult to observe in wild life animals or in this aspect less characterized species. Diffuse deposits of Aβ were observed in the parietal cortex of dolphins and more compact senile plaques in their cerebellum (Di Guardo, 2018). Sheep and goats (Braak et al., 1994; Nelson et al., 1994; Nelson and Saper, 1995), cheetah (Serizawa et al., 2012), bison (Härtig et al., 2000, 2001), bears (Cork et al., 1988) and most species of nonhuman primates (Schultz et al., 2000a, b; Rosen et al., 2008; Oikawa et al., 2010; Perez et al., 2013, 2016; Rodriguez-Callejas et al., 2016; Reid et al., 2017) also develop amyloid plaques and/or harbor phosphorylated TAU laden neurons, but most of them do not have NFTs present in their central nervous systems (as reviewed in Youssef et al., 2016). However, AT8-immunostained pTAUSer202 and pTAUThr205 were found in the brains of brown lemur, rhesus monkey, baboons, rabbit, guanaco, and bison (Härtig et al., 2000). Furthermore, NFTs were revealed by Gallyas staining in aged bison (Härtig et al., 2001).

There have been speculations on the reasons for the lack of NFTs in the canine brain and in the brains of other animals (Kuroki et al., 1997; Youssef et al., 2016). First possibility is the dissimilarity between canine TAU protein sequence in comparison to human’s. Secondly, the lifespan of dogs might be too short to develop NFTs, as Aβ deposition precedes NTFs formation. Thirdly, although the amyloid protein sequence is highly conserved between species, its N-terminal part is not, which might influence TAU phosphorylation and its subsequent aggregation into NFTs. Therefore, it seems that only humans develop the full blown AD pathology with amyloid plaques, NFTs and dementia. There have been few reports of possible AD in aged great apes (Rosen et al., 2008; Perez et al., 2013, 2016), although also in these the TAU pathology was very limited. Several reasons for this exceptional vulnerability of humans to develop AD have been proposed (Arendt et al., 2017; Walker and Jucker, 2017). Predisposition to develop AD might arise as early as during embryonic development over the period of neurogenesis, this was inferred from the modular deposition of AD related cerebral deposits which mirrors the formation and migration of neurons and on a larger scale development of brain regions (Arendt et al., 2017). In humans, the relative brain size is much larger than in any other animal and this expansion is due to longer duration of brain development as well as higher number of neuronal progenitor cell division cycles, the latter giving rise to genomic aberrations, which in turn can be the origin of AD (Arendt et al., 2017). Furthermore, the neurons display genomic mosaicism, which was detected in neurons of approximately 10% of normally aged people. These neurons were shown to preferentially undergo apoptosis in AD (Arendt et al., 2010). The extent of neuronal genomic mosaicism is brain region specific and correlates with differential vulnerability to neurofibrillary pathology (Arendt et al., 2015). Moreover, the pathological TAU conversion might commence as early as in children (Braak and Del Tredici, 2012), although it has not been firmly established if these processes are transient in nature. The canine model would therefore serve as an accessible and suitable model to further delineate why, except for humans, are other species less susceptible to develop tauopathies.

### The Neuropathology of Cerebral Blood Vessels

In patients with AD, accumulation of Aβ is often observed in the walls of blood vessels in the brain. This is called CAA and is caused by pathological deposits of Aβ and other proteins in the cerebral arterioles and capillaries of the leptomeninges and cortex. It is considered as an early and integral part of AD pathogenesis and the prevalence of CAA in AD is over 70% (Attems, 2005). CAA is a risk factor for hemorrhagic stroke and contributes to AD dementia. Atherosclerosis may also cause cerebrovascular dysfunction that may lead to cognitive decline as well as stroke (Shabir et al., 2018). The accumulation of amyloid in the walls of blood vessels is common in elderly people, patients with CAA and in almost all patients with AD (Kumar-Singh, 2008). The most CAA affected brain regions are the leptomeninges and frontal and temporal cortices (Borràs et al., 1999) with Aβ deposits in arteries, arterioles, capillaries, venules, and veins. The CAA disease progresses from mild, to moderate to severe stage, this is from almost intact blood vessels with minor Aβ deposits to extensive vascular Aβ load, which disrupts vessel architecture and exacerbates microangiopathies, microaneurysmal dilatation, and fibrinoid necrosis (Revesz et al., 2002).

**FIGURE 3:**
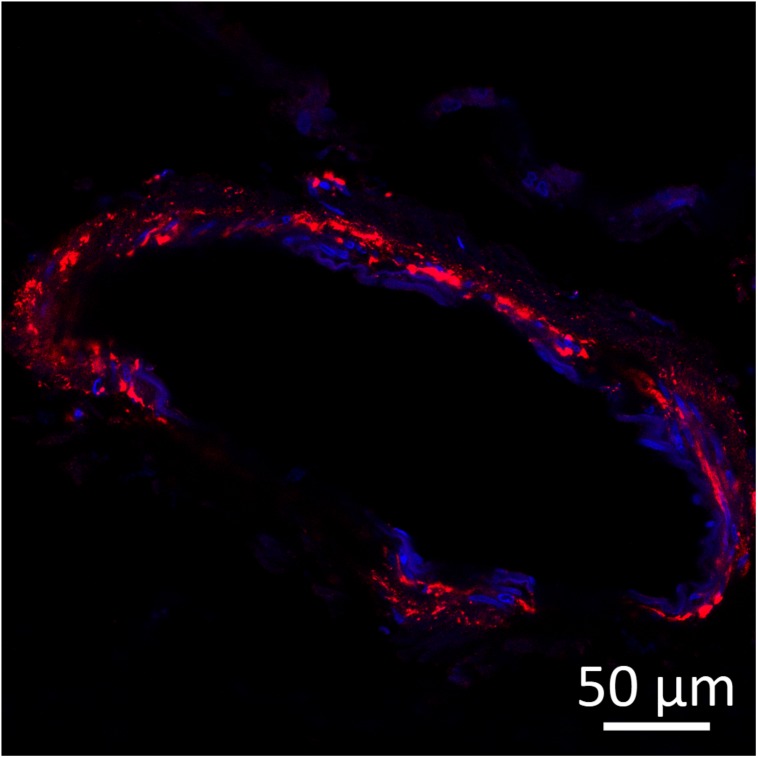
Amyloid beta (red) detected by immunofluorescence staining in the wall of a leptomeningeal blood vessel in the frontal cortex from a 15-years-old Pit Bull Terrier. Nuclei were counterstained with DAPI (blue). The antibody employed is the same as in Figure 2. Original microphotograph made by the authors.

In aged dogs Aβ deposits are detected both in the brain parenchyma and in the walls of cerebral blood vessels (Ishihara et al., 1991; Uchida et al., 1992; Borràs et al., 1999; Colle et al., 2000; Brettschneider et al., 2015; Schmidt et al., 2015; Ozawa et al., 2016; Nešić et al., 2017; Rusbridge et al., 2018), further suggesting similarities between AD in humans and CCD in dogs (Figures 2, 3). The amyloidotic blood vessels in the brains of dogs were observed by Congo red stainings as well as by antibodies directed against Aβ. Vascular Aβ deposition mainly consists of Aβ_40_ in humans, but both Aβ_40_ and Aβ_42_ were detected in dogs (Kumar-Singh, 2008; Chambers et al., 2012). In canine CAA, the most affected region is the frontal cortex, where Aβ is often present in microvascular parenchymal lesions in elderly dogs. In dogs with CCD an overlap between the density and anatomical distribution of CAA and parenchymal plaques was observed (Nešić et al., 2017). The CAA consistently increased with age, but did not correlate with the CCD score (Ozawa et al., 2016). Intracerebral hemorrhages have also been described in association with CAA in dogs (Uchida et al., 1990). A recent case reported multiple infarcts and brain hemorrhages in a dog with CAA and CCD, with cerebral and vascular Aβ deposits (Rodrigues et al., 2018). Pathological hallmarks of CCD and AD are summarized in Table 2.

**TABLE 2:** Pathological hallmarks of CCD and/or AD.

**Abnormality**	**CCD**	**AD**
Cognitive decline	+	+
Brain atrophy	+	+
Neuronal damage and death	+	+
Aβ accumulation in brain parenchyma	+	+
Diffuse Aβ plaques	+	+
Dense-core Aβ plaques	−	+
Aβ accumulation in blood vessel walls (CAA)	+	+
Neurofibrillary tangles (NTFs) formation	−	+
Microglial dysfunction	+	+
Astrocyte dysfunction	+	+
Astroglial hypertrophy and hyperplasia	+	+
Oxidative brain damage	+	+
Mitochondrial dysfunction	+	+
Cholinergic dysfunction	+	+
Impaired neuronal glucose metabolism	+	+

## Genetics

The incidence of human neurodegeneration and associated dementias is sporadic and is only rarely due to hereditary changes that are linked with genetic mutations. The majority of AD cases are sporadic late-onset (LOAD) with an unknown etiology (Cacace et al., 2016). The other rare AD form is genetically determined familial AD. Known specific gene mutations are the root cause for the development of familial form of AD (Selkoe, 1997). Most of the patients with early onset, familial form of AD have mutations in one of the two presenilin proteins (PSEN1 and PSEN2) (Bekris et al., 2010), which are proteins with a role in Aβ generation. Mutations also occur in *APP* gene (Selkoe, 1997). Polymorphism in APOEε4 allele of apolipoprotein E gene (*APOE*) is the major genetic risk factor for LOAD along with polymorphism in *TREM2* gene, which has a role in maintaining normal immune functions in the brain (reviewed in detail in Wolfe et al., 2018). In population studies, several genetic loci have been identified as risk factors for AD (e.g., *CLU, CR1, APP, PICALM, BIN1, ABCA7, MS4A, MEF2C CD33, EPHA1, CD2AP, APOE, TRIP4, TREM2, SORL1*), although the connection between genes in these loci and pathogenesis of AD has not been established (Ashford, 2004; Karch et al., 2012; Mäkelä et al., 2018). Interestingly, the *APOE4* polymorphism is unique to humans and its evolvement from the presumably ancestral primate form might have been instrumental for the AD pathogenesis (Mahley and Rall, 1999; Walker and Jucker, 2017).

Some presenilin mutations and mutations in *APP* have been connected to CAA (Hendriks et al., 1992; Grabowski et al., 2001; Rossi et al., 2004; Bugiani et al., 2010; Sellal et al., 2017) and also a polymorphism in the neprilysin gene was associated with CAA (Kumar-Singh, 2008), although the exact connections between these mutations and AD/CAA symptoms and progression are not yet understood. Furthermore, APOEε4 was shown to be a risk factor for CAA and both APOEε2 and ε4 alleles are associated with more severe forms of CAA (Nelson et al., 2013; Yu et al., 2015).

No mutations in specific genes have been reported in dogs with CCD so far. The only neurodegenerative disease in dogs that has been linked to specific mutation is degenerative myelopathy (DM), a condition caused by both demyelination and axonal loss in the canine spinal cord. In dogs with this disease, mutations have been found in a gene SOD1, coding for superoxide dismutase enzyme (Nakamae et al., 2015; Kobatake et al., 2016). Interestingly, this gene is in human patients associated with some forms of familial amyotrophic lateral sclerosis (Rosen et al., 1993). Beside mutations in SOD1 gene, risk for developing DM in dogs, as well as onset of disease, seem to be partially modulated by gene encoding SP110 nuclear body protein (SP110) at least in Pembroke Welsh Corgis (Ivansson et al., 2016). No other mutations or disease modifying loci related to neurodegeneration have been detected in dogs so far and thus, little is known about the potential genetic risks or genetic predisposition for CCD and CAA.

## Biomarkers

Neurodegenerative diseases are particularly challenging diseases, as they are difficult to diagnose in the initial stages. Although many years of research have been devoted to the identification of suitable biomarkers, preferably in blood, that would allow early diagnosis or even prediction of AD in humans, such markers remain elusive. Numerous candidates have been identified in both blood and CSF, but none of the markers identified so far have been used routinely in the clinics. CSF levels of Aβ, total TAU and hyperphosphorylated TAU are the most often monitored variables, along with PET molecular imaging of amyloid and TAU deposition, in diagnosis of early stages of AD (McKhann et al., 2011).

In dogs there are no biological markers that would allow accurate and early diagnosis of CCD. In most cases assessment of cognitive functions through several neuropsychological tests and excluding other conditions with overlapping symptoms is sufficient to confirm diagnosis when the disease has progressed, but markers for detecting disease in early stages would be very useful in veterinary medicine.

In dogs with CCD, plasma Aβ_42_, a longer Aβ isoform which is more fibrillogenic and associated with disease, was monitored as a biomarker potentially linked to CCD (González-Martínez et al., 2011; Schütt et al., 2015). One study showed highest Aβ_42_ plasma levels in younger healthy dogs, and significantly higher Aβ_42_ plasma levels in mildly cognitively impaired dogs in comparison to severely impaired dogs (González-Martínez et al., 2011). However, another study reported the opposite with highest Aβ_42_ plasma levels in CCD dogs (Schütt et al., 2015). Plasma ratio of Aβ_40_ and Aβ_42_, was shown to be similar in human AD and CCD as in healthy individuals (Mayeux et al., 2003; Lopez et al., 2008; González-Martínez et al., 2011) although in some studies decreases in plasma Aβ_42_ were an indicator of faster progression of CCD and AD (Pesini et al., 2009; González-Martínez et al., 2011). This points to a possible disease mechanism wherein aggregation and accumulation of Aβ in the brain results in lower levels of Aβ in plasma and CSF. A more recent human study showed decrease in plasma Aβ during the dementia stage of AD and increased levels of Aβ in plasma during vascular disease (Janelidze et al., 2016).

In healthy aged dogs with age related Aβ deposits, a decrease in levels of Aβ_42_, but not Aβ_4__0_ was detected in CSF (Head et al., 2010). Similarly, in elderly people Aβ_42_ has been monitored in CSF and the decrease in Aβ_42_ is suggested as biomarker for Aβ deposition in the brain and has been observed alongside brain atrophy (Fagan et al., 2009; Racine et al., 2016). Although CSF Aβ content decreased in the aging dog (Head et al., 2010), high levels of Aβ in the CSF of young and middle-aged dogs also correlated with impaired learning (Borghys et al., 2017). This infers high CSF Aβ levels in younger dogs, which are not likely to harbor depositions of amyloid in their brains yet, as an early biomarker for the development of cognitive impairment. One study also reported an increase of lactate, pyruvate and potassium concentrations in CSF of dogs correlating with severe cognitive impairment (Pugliese et al., 2005), but this has not been firmly established or confirmed. Therefore, there are no biomarkers available to monitor and predict CCD progress in dogs. Future studies aiming to develop such biomarkers would be needed, hoping to provide biomarkers for early detection of this disease.

## Diagnosis of CCD

Although CCD is highly prevalent the disease is severely under-diagnosed, affecting a growing population of aged dogs. The diagnosis of CCD is a diagnosis of elimination. The illness exacerbating symptoms, commonly also observed in CCD, must be excluded, such as brain tumors, hypertension, other neurological conditions, metabolic and hormonal imbalances, etc. Screening and diagnosis of CCD is primarily based on observation of clinical signs which are summarized by the acronym DISHAA [Disorientation, altered social Interactions, altered Sleep–wake cycles, House soiling and loss of other learned behaviors, altered Activity levels and increasing Anxiety (Ruehl et al., 1995; Neilson et al., 2001; Azkona et al., 2009; Rosado et al., 2012; Fast et al., 2013b; Madari et al., 2015)]. Sleeping during the day and restless at night, decreased interaction, disorientation at home and anxiety are common symptoms (Fast et al., 2013b).

The diagnosis depends largely on the owners and the veterinarians to observe and diagnose the disease, which is in most cases overlooked and symptoms attributed to the aging of dogs. To facilitate the detection of CCD, veterinarians can use a screening questionnaire that includes a list of possible signs. Several questionnaires are available and based on the scores the stage of dog’s cognitive decline can be identified (Colle et al., 2000; Neilson et al., 2001; Osella et al., 2007; Azkona et al., 2009; Golini et al., 2009; Salvin et al., 2011; Landsberg et al., 2012; Rosado et al., 2012; Fast et al., 2013b; Madari et al., 2015). Madari et al. (2015) have proposed criteria for discrimination of three stages of the disease, these are mild cognitive impairment, moderate cognitive impairment, and severe cognitive impairment. The severity and progression of CCD disease is identified by Canine Dementia Scale (CADES), which contains 17 items distributed into four domains related to changes in dogs’ behavior (spatial orientation, social interactions, sleep–wake cycles, and house soiling) (Madari et al., 2015). The Canine Cognitive Dysfunction Rating Scale (CCDR) is another questionnaire (Salvin et al., 2011), which is comprised of 13 behavioral items distributed into four domains, namely orientation, memory, apathy, impaired olfaction, and locomotion (Salvin et al., 2011). The cognitive deficits in dogs with CCD with regards to affected brain regions are summarized in Table 1. Cognitive impairment parallels the symptomology of AD. Performance on tasks involving complex learning and working memory are impaired first, along with executive function, visuospatial ability and complex learning deficits, with disease progression impairments in discrimination learning and behavioral changes are common (Neilson et al., 2001; Tapp et al., 2003; Studzinski et al., 2006; Madari et al., 2015).

MRI diagnosis is only rarely performed in dogs, due to possible complications during anesthesia and cost restraints. In dogs with CCD, as in humans with AD, MRI shows brain atrophy and include ventricular enlargement as well as widened and well-demarcated cerebral sulci. Measuring the thickness of the interthalamic adhesion in CCD was employed as a parameter for quantifying canine brain atrophy (Hasegawa et al., 2005; Noh et al., 2017). In demented canine brain MRI can be a useful tool to detect other abnormalities of the brain, possibly causing dementia such as leukoaraiosis (periventricular white matter hyperintensities) and brain microhemorrhages (Dewey et al., 2019). In human brain imaging a variant of amyloid-binding histological dye Thioflavin T, [^11^C]PiB, has been used as a tracer to detect Aβ using PET (positron-emission tomography) scans. In dogs this compound failed to detect the full amyloid load in the brain, established by *ex vivo* histopathological investigation (Fast et al., 2013a).

In general, the diagnosis of human AD is set similarly. First by ruling out other possible causes for symptoms and then by detection of CSF and plasma biomarker levels, tests of memory, problem solving, attention, counting, and language, which can be followed by MRI and PET scans (Hane et al., 2017). As in dogs, the cause of dementia can only be confirmed with certainty postmortem.

## Treatment and Drug Development

Current treatment approaches for AD in humans are focused on helping people maintain mental function, manage behavioral symptoms, and slow or delay the symptoms of the disease. Unfortunately, there is no effective treatment for AD. Drugs that are used today in the management of AD can only alleviate the symptoms, and even that only temporarily. Cholinesterase inhibitors (donepezil, rivastigmine, and galantamine) have been approved for the management of AD in humans. Drugs acting as acetylcholinesterase inhibitors reduce the activity of the acetylcholine esterase which degrades acetylcholine, thus increasing the amount of acetylcholine available in the brain and therefore stimulate brain cells which receive more synaptic inputs (reviewed in Winblad et al., 2016). This very unspecific treatment can slow the disease progression for 6–12 months, and even achieve temporary improvement as shown for donepezil (Birks and Harvey, 2018), but does not cure the disease which will eventually progress. Another drug used for the treatment of AD is memantine that acts on the glutamatergic system by blocking NMDA (*N*-methyl-D-aspartate) receptors. A recent random-effects network meta-analysis of 41 randomized controlled trials reveled the most suitable dosages of cholinesterase inhibitors and memantine to treat patients with mild, moderate and severe AD (Dou et al., 2018). Although these pharmacological interventions have beneficial effects on cognition, function and global clinical impression, these treatments do not alleviate the neuropsychiatric symptoms (Dou et al., 2018). Unfortunately, there have been numerous clinical trials with many candidate drugs, but most of these had negative outcomes (Cummings et al., 2018).

### Treatment of Canine Dementia

Current treatment options for CCD target prevention, slowing and/or improving the cognitive decline in dogs. Some drugs or food supplements are available for senior dogs and might act neuroprotective. Some enhance the blood flow into the brain, others work as antioxidants and more effort is now directed to slowing the progression of the disease instead of providing only symptomatic treatment. One commonly prescribed drug for cognitive impaired dogs is selegiline, which acts as an inhibitor of monoamine oxidase B (MAOB), thus reducing degradation of several neurotransmitters in the brain, and may have neuroprotective effects on dopaminergic, noradrenergic and cholinergic neurons (Landsberg, 2005; Magyar, 2011). Another drug that is occasionally used is nicergoline, which increases the blood flow through the brain. It may enhance neuronal transmission and act neuroprotective, increase dopamine and noradrenaline turnover and inhibit platelet aggregation (Landsberg, 2005). Propentofylline also has a neuroprotective role as it inhibits the production of free radicals and reduces the activation of microglial cell, thus acting anti-inflammatory (Frampton et al., 2003). Antidepressants such as selective serotonin reuptake inhibitors fluoxetine and sertraline, amitriptyline, paroxetine and anxiolytics benzodiazepines, gabapentin, valproic acid and buspirone can also be used to treat the anxiety and aggression which may accompany CCD. Clomipramin is an antidepressant commonly prescribed for dogs with anxiety (Landsberg, 2005), but these are all symptomatic treatments and do not treat the disease itself. *S*-adenosylmethionine tosylate supplementation was reported to be safe and effective in improving signs of age-related mental decline in dogs (Rème et al., 2008).

There are also some nutraceutical preparations available for dogs, which are based on natural products and/or supplement formulations. Behavioral enrichment alongside with antioxidant-rich diet and exercise is an approach for maintaining cognitive function and slowing the progression of CCD in senior pets. As means of preventative intervention, aging beagles were fed a diet rich in antioxidant, which improved cognition, maintained cognition and reduced oxidative damage and Aβ pathology in treated dogs (Milgram et al., 2004; Dowling and Head, 2012). Another longitudinal survey in beagles looked at the proteomic changes following administration of antioxidant-rich diet in combination with behavioral enrichment (Opii et al., 2008). Following treatment, the levels of oxidative stress biomarkers decreased and the increased expression levels of Cu/Zn superoxide dismutase, fructose-bisphosphate aldolase C, creatine kinase, glutamate dehydrogenase and glyceraldehyde-3-phosphate dehydrogenase correlated with improved cognition (Opii et al., 2008). In addition, some other studies implicated the nutrition as cognition modifying factor in dogs (Araujo et al., 2005a; Siwak et al., 2005; Osella et al., 2007; Christie et al., 2009; Head et al., 2009; Snigdha et al., 2011, 2012; Katina et al., 2016; Chapagain et al., 2018), highlighting combination of nutraceutical supplements directed at several mechanisms of pathological aging, in combination with behavioral enrichment, as more effective (Araujo et al., 2005a, 2008). Dogs receiving both an antioxidant-rich diet and environmental enrichment showed increased levels of brain-derived neurotrophic factor (BDNF) mRNA when compared to untreated aged dogs. As a result of increase in BDNF mRNA, the cognitive performance improved and the amount of cortical Aβ deposits decreased (Fahnestock et al., 2012). Interesting is the finding that neuronal loss in the hippocampus, occurring in the aged dogs, could be partially reversed by more engagement with the dog, i.e., with stimulation of brain function (Siwak et al., 2000).

### Animal Models for Developing Novel Treatments

In comparison to transgenic mouse models, natural animal models better represent the pathophysiology of AD. Models of “physiologically” aged rats, degus and dogs are useful for studying mechanistic aspects of AD, which are also very valuable in the development of therapeutics that would alleviate age-related declines in cognitive function. Mouse models for AD research carry mutations, found in familial AD, and are artificially accumulating Aβ plaques and NFTs. Whether mice are good models have been thoroughly discussed elsewhere (Götz et al., 2018). Several drugs have cleared the amyloid load in mice but failed to do so in people with AD. Recently the genetic background and environmental factors have been demonstrated as the variability in AD development, which was partially recognized by incorporating genetic diversity into mouse models of AD (Neuner et al., 2018). Transgenic minipigs expressing *APP695* or *PSEN1* have also been developed but have not shown the histopathological nor the cognitive impairment signs (Holm et al., 2016). Therefore, natural animal models of species with spontaneously occurring neurodegeneration are potentially more useful in developing and testing novel treatments for such diseases.

To date, most of the drugs in development for AD treatment have been directed toward the removal of amyloid plaques or NFTs, not taking into the account the multifactorial causation of the disease. Several experimental drugs that have successfully removed plaques from mouse brains have not lessened the symptoms of AD in people. For instance, drugs acting as BACE1 (beta-site amyloid precursor protein cleaving enzyme-1) inhibitors had failed in Phase II/III clinical trials (Hawkes, 2017; Dobrowolska Zakaria and Vassar, 2018; Egan et al., 2018). A BACE1 inhibitor verubecestat successfully blocked the accumulation of amyloid protein in mice (Villarreal et al., 2017), rats and monkeys (Kennedy et al., 2016), but did not reduce cognitive or functional decline in patients with mild to moderate AD (Egan et al., 2018). Although decrease in Aβ biomarkers in CSF and brain has been noticed after treatment with BACE1 inhibitors, the failure to prevent cognitive decline might have been due to irreversible neurotoxic accumulation of Aβ that occurred prior to the start of the treatment. For this reason, focus is on the development of therapies that commence at presymptomatic stage (preclinical stage and the stage of mild cognitive impairment) although for this, development of novel, useful biomarkers, is also crucial.

As canine cognitive decline and human Alzheimer’s disease show neuropathological, cognitive and behavioral parallels, the testing of products for the treatment of AD in canine model could be useful to determine the efficacy of these compounds in humans, and also to develop novel therapeutic agents for the treatment of senior dogs. Several drugs have been tested in elderly dogs and their suitability and effectiveness correlated with results obtained in human trials, when available. Drugs tested in dogs are listed in Table 3.

**TABLE 3:** Pharmacological interventions tested in dogs with cognitive decline.

**Name/type**	**Mode of action**	**Testing in dog**	**Results/outcomes**	**References**
LY2886721	BACE1 inhibitor	Pharmacology study in dogs and clinical trial in healthy volunteers	Aβ lowering in plasma and CSF	May et al.,2015
Selegiline (L-deprenyl)	MAOB inhibitor	Longitudinal study	Higher life expectancy (cognitive status not monitored)	Ruehl et al.,1997
Selegiline (L-deprenyl)	MAOB inhibitor	Performance studies	Improved visuospatial working memory (in only a subset of dogs)	Head et al., 1996; Campbell et al., 2001; Studzinski et al.,2005
Atorvastatin	Reduction of Aβ and BACE1	Longitudinal study	Neuroprotective	Barone et al.,2012
Adrafinil	A wakefulness-promoting agent (eugeroic) with nootropic effects	Longitudinal study; pharmacological study	A significant increase in locomotion; improved learning; impaired working memory	Siwak et al., 2000; Studzinski et al.,2005
Ampakine	Positive modulator of AMPA receptors (enhance excitatory glutamatergic neurotransmission)	Pharmacological study	Decrease of performance accuracy; may have memory impairing effects	Studzinski et al.,2005
CP-118,954	Acetylcholinesterase inhibitor	Pharmacological study	Minimal cognitive enhancing effects	Studzinski et al.,2005
Phenserine	Acetylcholinesterase inhibitor	Pharmacological study; performance study	Enhancing effects on memory and learning; improved performance (only in a subset of dogs treated)	Studzinski et al., 2005; Araujo et al.,2011a
Donepezil	Acetylcholinesterase inhibitor	Performance study	memory enhancement	Araujo et al.,2011a
CNP520	BACE1 inhibitor	Dogs, human	Safe to use in dogs; tolerated in healthy humans; ongoing clinical trials	Neumann et al.,2018
Antioxidant-rich diet with cognitive enrichment	/	Dogs	Improved cognition	Opii et al., 2008; Dowling and Head,2012
Anti-Aβ immunotherapy	Passive vaccination with injections of antibodies against Aβ_42_	Dogs	Reduced amyloid plaques and reduced astrogliosis	Head et al., 2008; Neus Bosch et al., 2015; Davis et al.,2017

BACE1 is a protease that controls the formation of Aβ and most likely plays an important role in the development of pathogenesis in AD. The usefulness of BACE1 small-molecule inhibitor LY2886721 has been tested in a dog model and in humans in the first clinical phase (May et al., 2015). It significantly reduced plasma and CSF Aβ levels both in dogs and healthy volunteers (May et al., 2015). After administration of two BACE1 inhibitors (cyclic sulfoxide hydroxyethylamine NB-B4 and oxazine derivative NB-C8) a unique pattern of secreted Aβ peptides was observed in canine CSF (Mattsson et al., 2012). Besides the expected reduced levels of Aβ_40_ and Aβ_42_, reduced levels of Aβ_1–__34_ and increased levels of Aβ_5–40_ were detected, which were proposed as prognostic markers of BACE1 inhibition therapies (Mattsson et al., 2012). A recent survey, using BACE1 inhibitor CNP520, demonstrated reduced brain and CSF Aβ in rats, dogs, and reduced CSF Aβ in humans and was assessed to be well tolerated in healthy adults and further clinical trials are ongoing (Neumann et al., 2018). A cardiovascular disease drug, atorvastatin, has been investigated in a canine model of dementia whether it could reduce Aβ plaques, BACE1 protein levels and oxidative stress (Barone et al., 2012). It does offer some neuroprotective role through the up-regulation of enzyme biliverdin reductase-A (Barone et al., 2012). Selegiline (L-deprenyl), a type B monoamine oxidase inhibitor, has been reported to prolong the survival of aged dogs (Ruehl et al., 1997) and in some cases improve visuospatial working memory (Head et al., 1996). However, in subsequent studies only a small subset of dogs seemed to show improvement, pointing to a minor clinical relevance of selegiline administration (Campbell et al., 2001). Furthermore, several human AD trials provided no evidence of clinically meaningful benefits of selegiline administration for people with AD (Birks and Flicker, 2003). Interestingly, although selegiline was shown to be fairly ineffective in the treatment of CCD, it is still the only FDA-approved treatment for CCD.

Testing the same compounds in dogs and humans provided similar findings also in other studies. CP-118,954, an acetylcholinesterase inhibitor, showed minimal cognitive enhancing effects in dogs and human clinical trials were discontinued (Studzinski et al., 2005). Donepezil administration enhanced memory in dogs (Araujo et al., 2011a). Phenserine, an acetylcholinesterase inhibitor which also inhibits synthesis of Aβ and acts as a cognitive enhancing therapeutic, improved learning and working memory in geriatric dogs (Studzinski et al., 2005; Araujo et al., 2011a). Its administration showed similar results in Phase II human clinical trials, suggesting its effectiveness in improving memory in AD patients, but in Phase III human clinical trial, with patients with mild to moderate AD, no significant differences between the phenserine treated and placebo groups were noticed and the trial was discontinued (Thatte, 2005). There has been further discussions if this trial, and several other AD trials, failed due to procedural errors rather than due to a lack of drug efficacy (Winblad et al., 2010). In more recent studies patients with mild AD showed improvement in cognition following phenserine treatments, this was mirrored also by an increase in CSF Aβ_40_ (Kadir et al., 2008; Nordberg et al., 2015)_._ There is another mode of action of phenserine in AD, namely inhibition of neuronal self-induced preprogrammed cell death, which further clinical trials might pursue (Becker et al., 2018). Ampakine (drug that alters the glutaminergic system) also failed in human AD trials and in dogs with age-associated memory disorder and dementia showed insignificant memory-enhancing effects after treatment with ampakine (Studzinski et al., 2005). Dogs, treated with adrafinil, a mild central nervous system stimulant used to relieve excessive sleepiness and inattention in elderly patients, were more attentive (Siwak et al., 2000). Cholinergic hypofunction might play a role in age-dependent cognitive decline in dogs (Araujo et al., 2011b), this was determined by administration of muscarinic cholinergic receptor antagonist scopolamine, which induces transient memory impairment. Similar findings were presented for aged and demented humans (Schliebs and Arendt, 2011). All these suggest that dogs might be a very good preclinical model for developing and testing new drugs for AD in humans.

Besides BACE1 and cholinesterase inhibitors several attempts have been made to develop immunotherapy treatments directed against Aβ or TAU (Bittar et al., 2018). A range of TAU-targeted immunotherapies have entered clinical development (recently reviewed in Novak et al., 2018). Clinical trials in patients with AD, which were administered Aβ peptide with conjugate to stimulate the immune response (i.e., active immunization) or anti-Aβ antibodies (i.e., passive immunization) have shown modest results and also serious side effects (reviewed in detail in Wisniewski and Drummond, 2016).

In dogs, Aβ immunotherapy could reduce the presence of amyloid plaques and astroglial reaction in aged individuals (Neus Bosch et al., 2015). A vaccine directed against fibrillary Aβ (anti-Aβ_42_) reduced the presence of Aβ plaques in canine prefrontal cortex and improved the function of the frontal cortex, thus resulting in cognitive benefits (Head et al., 2008). Anti-Aβ_42_ vaccination of dogs with CCD, in combination with behavioral enrichment, resulted in reduced presence of Aβ plaques in the brain, a significant maintenance of learning abilities over time and cognitive maintenance with no improvement in cognition (Davis et al., 2017). This anti-Aβ vaccine, however, also increased the frequency of brain microhemorrhages (Davis et al., 2017) which is commonly observed in the development of these immunotherapies also in human trials and could be very serious side effect. Similarly to pharmacological compounds, immunotherapies in both dogs and humans have provided modest improvements at most, yet similarities between human and canine patients again highlight the parallels between diseases and usefulness of dogs as a preclinical model for AD.

## Conclusion

Risks for age-related AD and CCD are a complicated interplay between aging, genetic risk factors and environmental influences. CCD in dogs is similar to human AD with respect to APP processing, amyloid plaque deposition and cognitive dysfunction. As dogs age, they have been shown to accumulate amyloid plaques, but, dissimilar to human AD patients, dog brain rarely contains NFTs. Aβ peptide accumulates in the canine brain extracellular space in the form of soluble oligomers, fibrils and Aβ plaques. Its toxic accumulation is believed to be responsible for the neuronal dysfunction and degeneration although the severity of the disease does not always correlate with amyloid burden, but might correlate with toxic fibril polymorphs.

Dogs over 7–8 years seem to be interesting, naturally occurring model organism for aging/dementia that fairly faithfully recapitulate human disease. They are potentially much more useful as rodent models as in laboratory rodents neurodegenerative diseases do not occur spontaneously. Furthermore, genetically modified mice used in research often show very different neurodegeneration courses, as seen in humans. Dogs also spontaneously develop vasculopathies, such as CAA, and perhaps vascular dementia although this has not yet been truly studied, and are therefore also valuable as models to decipher the age and/or dementia related cerebrovascular changes that often accompany or even precede neurodegeneration in human patients.

There are no effective treatments for neurodegenerative disorders, practically all currently available treatments are symptomatic. This is partially related to the poor understanding of the pathogenesis of these diseases, and partly due to the lack of good animal models. Cholinesterase or secretase inhibitor therapy or immunotherapy has been attempted in dogs with CDD, with overlapping results to human AD trials. The neuroprotective effects of cognitive enrichment, alongside with antioxidant-rich diet, show benefits in managing the disease progression and severity in both humans and dogs. Similar results with various drugs do suggest that dogs are useful model to study both pathogenesis and novel treatments for AD in the future.

Taken together, animal studies are important to dissect the molecular and cellular processes specific to AD pathology. Dogs with CCD not only develop isomorphic changes in human cognition and brain pathology, but also accurately predicted the efficacy of known AD treatments and would be therefore good models for testing new substances that affect the lowering of Aβ levels or the reduction or degradation of aggregates associated with AD. This would benefit human patients, but could also help dog patients as any successful treatments could be also introduced into clinical veterinary medicine with the aim to successfully treat this debilitating disease in canine patients.

## Author Contributions

Both authors have made a substantial contribution to this work.

## Conflict of Interest Statement

The authors declare that the research was conducted in the absence of any commercial or financial relationships that could be construed as a potential conflict of interest.
